# Absolute calibration methodology for non-uniform uranium and matrix distributions in large barrels of uranium-bearing solid waste

**DOI:** 10.1038/s41598-024-78701-y

**Published:** 2024-12-31

**Authors:** K. M. El-Kourghly

**Affiliations:** Nuclear Safeguards and Physical Protection Department, Egyptian Atomic Energy Authority (EAEA), Cairo, Egypt

**Keywords:** Uranium-bearing solid waste, Segmented Gamma scanner, Monte Carlo simulation, Energy science and technology, Physics

## Abstract

The effective implementation of domestic and international safeguards necessitates verification techniques for Nuclear Materials (NM). Even in the case of very small quantities of NMs, accounting for and analyzing such traces can provide insights into the mass balance of NMs and/or state activities, ensuring consistency in state declarations. This paper proposes and benchmarks an absolute calibration methodology for estimating the uranium–mass content in large-volume barrels (200 L). These barrels may be generated during the operation and decommissioning of bulk handling facilities by accumulating low-density scarab contaminated with NMs. The method relies on the mathematical calibration of a High Purity Germanium (HPGe) detector efficiency against non-uniform uranium and matrix distributions, assuming that the non–uniform distribution can be approximated as a uniform one for low–density matrix materials. The peak efficiency is calculated for different numbers of point sources 1–30 likely distributed inside a simulated barrel while it rotates around the axis of symmetry. The proposed method is benchmarked by comparing the calculated peak efficiency of randomly distributed uranium sources with the homogeneous distribution. Furthermore, a comparison with experimental measurements is conducted to validate the proposed method. Results show that the proposed calibration method considering either random or homogeneous source and matrix distributions in large volume barrels can be used for estimating the uranium mass content in NSW with an accuracy of approximately 12 %.

## Introduction

Nuclear Solid Waste (NSW) generated throughout the various stages of the nuclear fuel cycle, including fuel fabrication, may contain small quantities of NMs. Despite their relatively low concentration and purity, these quantities could be significant in the realm of nuclear non-proliferation. Hence, the potential to recover these materials from waste cannot be disregarded. However, the associated risk posed by the waste will be contingent on the concentration of NMs within it and the physical characteristics of the waste.

Characterizing NSW and its constituent materials, including container material, volume, physical and chemical form, irradiation status, fissile isotopic mass, type, enrichment, as well as the total NM content, is imperative for submitting comprehensive reports to the International Atomic Energy Agency (IAEA) under the application of the Comprehensive Safeguards Agreement (CSA).

These NSW may be contained in large plastic barrels with a volume of approximately 200 liters. Their containment consists of low–density scrap materials (e.g., papers, wipes, plastics, filters,...etc.) contaminated with NMs. These wastes exhibit non–uniform distributions of NMs and matrix, which poses challenges for the accurate characterization and/or verification of the NM content. The segmented $\gamma$–Scanner (SGS) can be employed for the characterization and/or verification of NMs in matrices of low–density materials. The SGS provides quantitative corrections for material attenuation on a segment–by–segment basis with the assistance of a transmission source. This technique has been employed by Refs.^1–5^ for quantifying NMs with various equipment configurations. Despite its high accuracy, this technique necessitates a standard drum and a high–activity transmission radioactive source, typically in the range of millicuries (*mCi*), which could pose safety issues. Additionally, the high cost renders the system less accessible to a wider users.

The relative calibration technique proposed by El-Kourghly et al.^6^ could be employed for the characterization of NSW. The calibration curve is generated using a simulated barrel filled with a varied distribution of Low Enriched Uranium (LEU) samples in powder form. While providing accurate results, standard sample preparation necessitates safety precautions to avoid potential contamination with LEU. Moreover, the backing material may vary from one barrel to another, making the construction of different calibration curves to correct for all barrels complex, tedious, and time–consuming.

The work described here aims to build on long research and development towards NSW characterization, as explored by El-Kourghly et al.^6^. Specifically, this work investigates replacing SGS and relative calibration techniques with an absolute calibration methodology for characterizing irregular uranium and matrix distributions in operational waste containing NMs. The objective is to address safety concerns associated with the presence of a transmission radioactive source in SGS systems, potential contamination during the preparation of U–powder samples, and to provide flexibility in constructing an efficiency calibration curve that accommodates different packing conditions of the containers. We anticipate an accuracy of less than 15 % for this method; thus, it is designed to evaluate its effectiveness in fulfilling national and international obligations concerning the declaration of NMs.

## Methods description

The proposed methodology relies on mathematical peak efficiency calibration using the general Monte Carlo N-Particle (MC) code. Throughout the current investigation, the following factors and assumptions are considered:

- Matrix and ^235^*U* isotopic material distributions are irregular;
- Barrels contain materials with relatively low self–attenuation coefficients against $\gamma$–radiation;
- An irregular distribution of ^235^*U* and matrix inside a rotating barrel is considered approximately equal to a homogeneous distribution for low–density filled materials;
- Errors arising from ^235^*U* distribution can be mitigated by rotating the assayed barrel around its axis of symmetry during the calculation.

The proposed method is achieved through the following steps:

- Validation of the assumption that a non–uniform ^235^*U* and matrix distribution is approximately equal to a homogeneous one for low–density filled materials.

The aforementioned assumption is benchmarked by simulating a barrel filled with low-Z materials (plastics, papers, and tissues). The peak efficiency is calculated using MC code, assuming a different number of point sources randomly distributed inside the barrel. The peak efficiency of an HPGe detector is calculated as the barrel rotates around its axis of symmetry from 0 to 355 degrees with a 5-degree step size. The average peak efficiency is then compared with the homogeneous one to assess the reliability of the proposed assumption.

- A different number of point–like sources (1-30) are randomly distributed inside the simulated barrel to establish the mathematical calibration curve.
- The accuracy of the method is estimated by using biased randomly distributed point-like sources in comparison with the homogeneous distribution.
- The validation of the proposed method is conducted through a comparison with experimental measurements^7^.

## Monte Carlo calculations

The characteristics and specifications of an HPGe detector with crystal size of 2.6 cm radius, 3.1 cm height and 14 $\%$ relative efficiency at the  1332 keV $\gamma$–energy line in ^60^Co decay, based on manufacturer data, are modeled using MCNPX^8^ utilizing the F8 tally for pulse height tally. This tally is a built–in function used to simulate the detector response function by estimating the energy deposited in a cell over an entire particle history.

### Model optimization

**Fig. 1:**
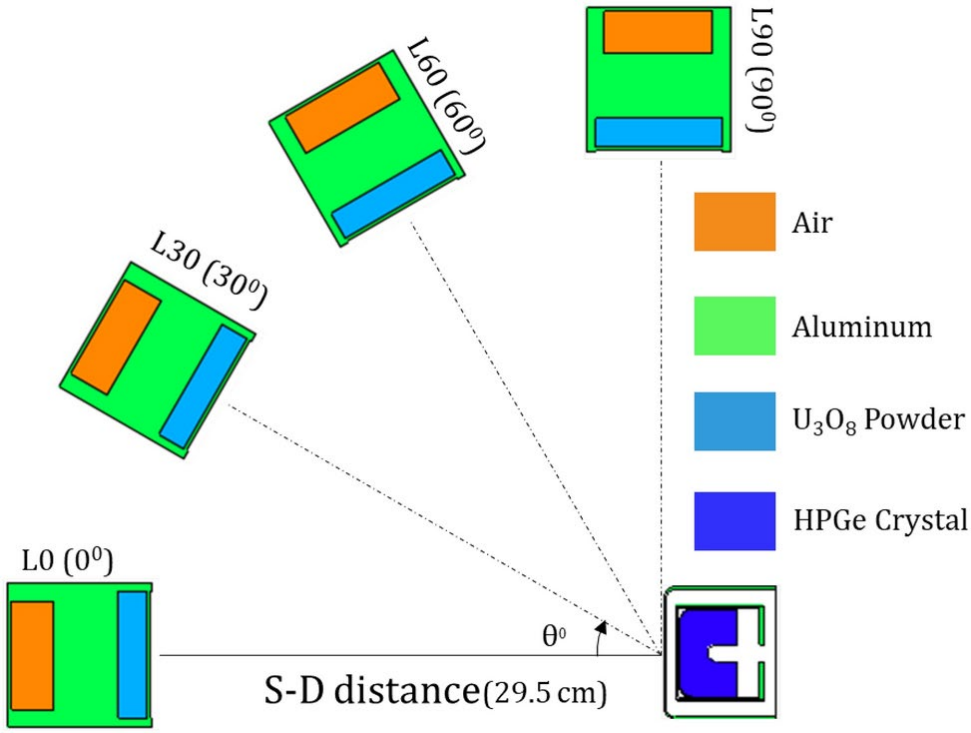
S-D configurations for model optimization.

To optimize the detector’s model, a Standard Nuclear Material (SNM) is located at various angles with respect to the detector’s symmetry axis and at a distance of 29.5 cm from the detector’s front facet, as illustrated in Fig. 1. The angles include $0^{\circ }$ (L0), $30^{\circ }$ (L30), $60^{\circ }$ (L60), and $\perp$ to the detector central axis (L90). The peak efficiency of the 185.7 keV peak of ^235^*U* isotope is computed and compared to the experimentally estimated value. The thickness of the inactive Germanium layer is optimized using the trial-and-error method, adjusting it in defined steps until the optimal accuracy between the MC calculated and experimentally estimated peak efficiency is less than 1%.

### Validation of the proposed assumption

**Fig. 2:**
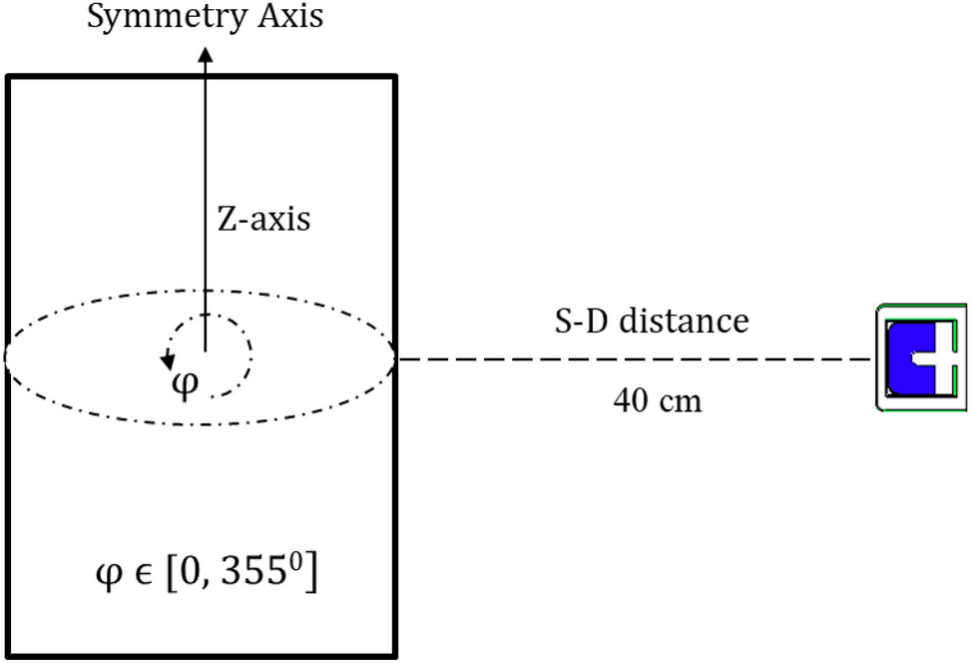
S-D configuration used for method validation.

The optimized MC model is utilized to verify the proposed assumption using a simulated 200 L barrel. The barrel is filled with low-density material to emulate real conditions. The calculation involves placing the barrel 40 cm away from the front facet of the detector, aligning the barrel’s axis of symmetry $\perp$ to that of the detector as shown in Fig. 2. The peak efficiency of the 185.7 keV peak of ^235^*U* isotope is determined under two conditions:

- Assuming a uniform distribution of the ^235^*U* isotope throughout the barrel volume;
- Considering various point sources of the ^235^*U* isotope randomly distributed throughout the barrel volume. The peak efficiency is calculated during the barrel rotation around it’s central axis of symmetry from $0-360^\circ$ with $5^\circ$ step size and then averaged.

The validity of the proposed assumption is confirmed by comparing the calculated peak efficiencies between random, and homogeneous distributions.

### Mathematical calibration curve

**Fig. 3:**
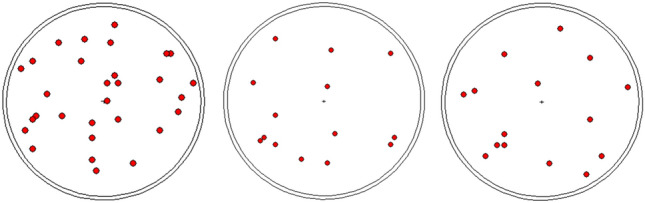
Examples of different number of point sources randomly distributed through the barrel top view.

**Fig. 4:**
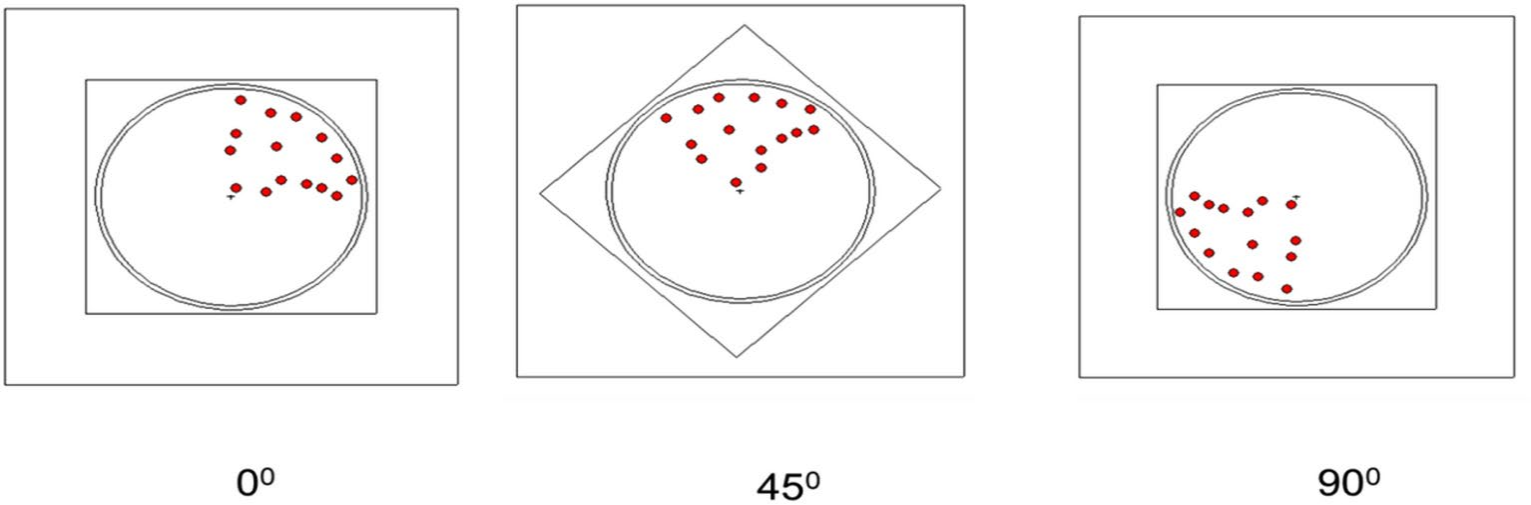
Example of a rotating barrel around its symmetry axis.

To establish the mathematical calibration curve, the S–D configuration shown in Fig. 2 is considered where, different point sources of ^235^*U*–isotope ranging from 1 to 30 are anticipated to be randomly distributed across the entire volume of the barrel as shown in Fig. 3. For each distribution the peak efficiency is calculated during the barrel rotation around it’s axis of symmetry in the range 0–${355}^0$ with ${5}^0$ step size as presented in Fig. 4. A total of 10 random distributions are taken into account for establishing the mathematical calibration curve.

The calculations are executed on a single-threaded Intel CORE 2 Duo processor with a clock speed of 2.66 GHz. Each simulation involves a total of $5 \times 10^7$ histories for all input files, and 720 input files are taken into account.

### Method accuracy

**Fig. 5:**
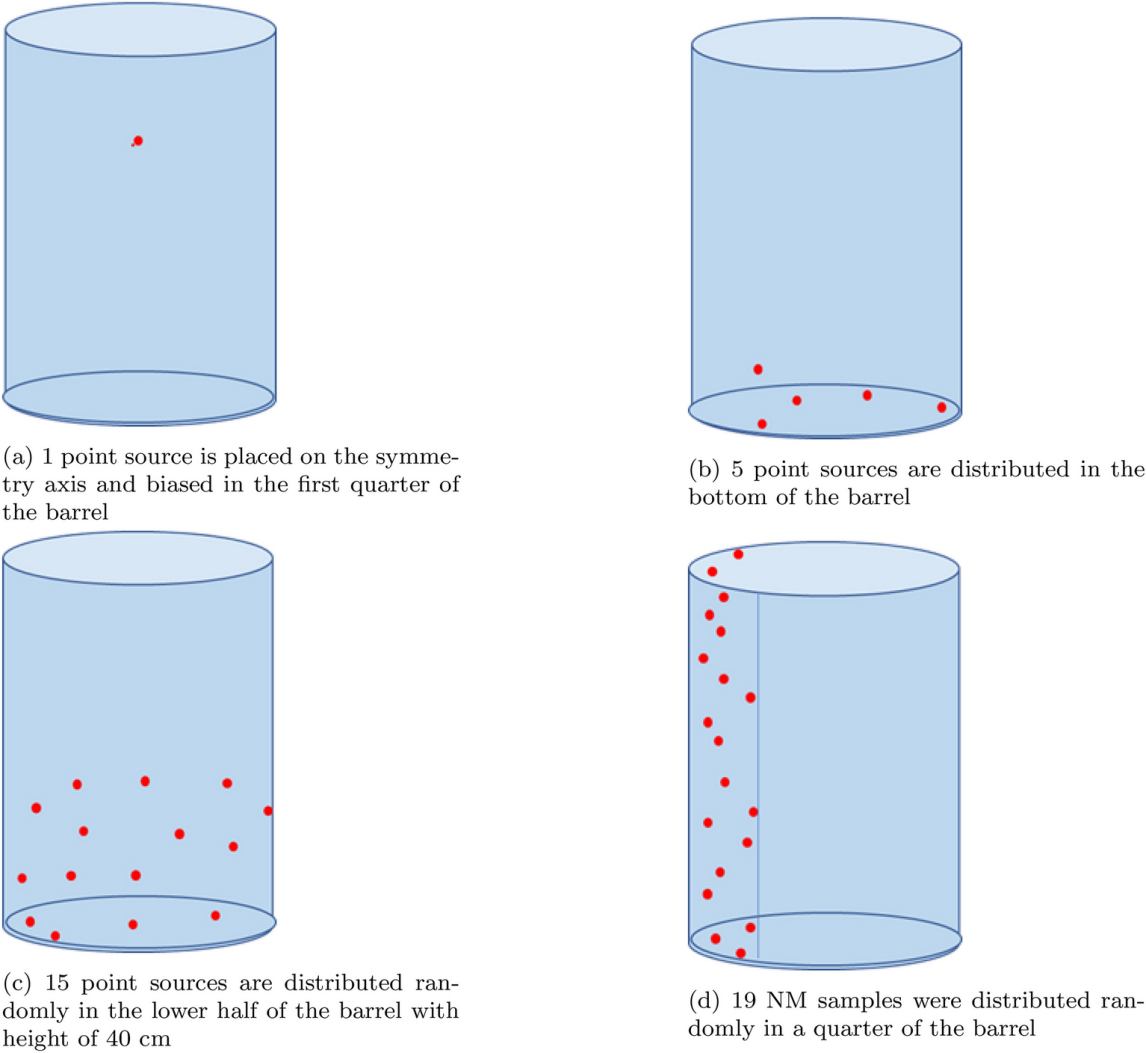
The different biased random distribution of of point sources through the barrel for method accuracy.

The accuracy of the proposed method is achieved through considering extreme cases where the NM distribution present in a specific sector of the barrel. For that a biased random distributions of point sources through a specific sector of the barrel are considered. A total number of four biased distributions (BD01, BD02, BD03, and BD04) are considered as shown in Fig. 5, and described as follows,

- BD01, 1 point source corresponding to 0.168 g ^235^*U* is placed on the symmetry axis and biased in the 1st quarter of the barrel Fig. 5a,
- BD02, 5 point sources corresponding to 0.84 g ^235^*U* mass are distributed in the Bottom of the barrel Fig. 5b
- BD03, 15 point sources corresponding to 2.52 g of ^235^*U* are distributed randomly in the lower half of the barrel with Height of 40 cm Fig. 5c,
- BD03, 19 point sources corresponding to 3.192 g of ^235^*U* are distributed randomly in a quarter of the barrel Fig. 5d,

A total of 288 MC input files are created to estimate method accuracy. The peak efficiency calculation for the considered distributions is conducted according to the description in Subsect. "Mathematical calibration curve".

## Experimental measurements

### Nuclear materials

- A cylindrical standard NM containing 4.46 % of ^235^*U*, specifically SRM 969-446-NBS 111, in the form of $U_3O_8$ powder, is utilized for optimizing the MC detector model. The NM is enclosed within a cylindrical aluminum can with dimensions of 8.898 cm in height and 8 cm in diameter.
- Thirty samples of Low Enriched Uranium (LEU), with an average composition of 19.75±0.2% ^235^*U*, are prepared. Each sample consists of $1 \pm 0.002$ g of $U_3O_8$ powder and is enclosed in sealed plastic bag. The powder is distributed in such away to minimize the self-attenuation factor. The entire preparation process is conducted inside a glove box to ensure safety precautions are met.

A barrel with dimensions of 23 “diameter x 38” length, has been prepared. This barrel is filled with a low-density material, and within it, LEU samples are randomly distributed throughout the barrel. The distribution involves varying the number of LEU samples, ranging from 4 to 30, throughout the entire volume of the barrel. Six different distributions (DR1, DR2,..., and DR6) are considered using 4, 7, 12, 20, 25, and 30 NM samples. These correspond to 0.168, 0.670, 1.171, 2.009, 3.353, 4.191, and 5.030 g of ^235^*U* mass, respectively.

### Instrumentation

Micro-Detective 200 HPGe Spectrometer^9^ of about 14% relative efficiency is used for count rate measurements. The spectrometer is equipped with Stirling-cycle cooler, and controlled using the $\gamma$–acquisition and data analysis software Mastero 32^10^.

The experimental measurements conducted elsewhere^6^ by placing the simulated barrel, with varying LEU masses and distributions, perpendicular to the symmetry axis of the detector and at a distance of 40 cm from the front facet of the detector. The choice of the S–D distance ensures an adequate solid angle coverage for the entire barrel. The detector is positioned at the midpoint of the assayed barrel. Figure 2 illustrates the experimental setup configuration, depicting the HPGe detector and the simulated barrel. Count rates are obtained using Maestro 32 software as the barrel rotates around its symmetry axis within the range of 0 to ${315}^0$, with a step size of ${45}^0$ as depicted in Fig. 4. A total of 7 experiments are conducted to validate the mathematical calibration curve.The measuring time for all these measurements is set at 900 s, and the dead time is approximately 1%.

## Results and discussion

This work establishes a mathematical calibration methodology intended for NM measurements in NSW. The approach assumes that the heterogeneous NM distribution transforms into a homogeneous one, especially applicable to relatively low–density matrix materials. This waste could be stored in 200 L capacity barrels, containing heterogeneous uranium and matrix distributions. To mitigate errors arising from the heterogeneous distribution of uranium, the barrels are rotated along their symmetry axes during calculation and measurements.

### MC detector model optimization

**Fig. 6:**
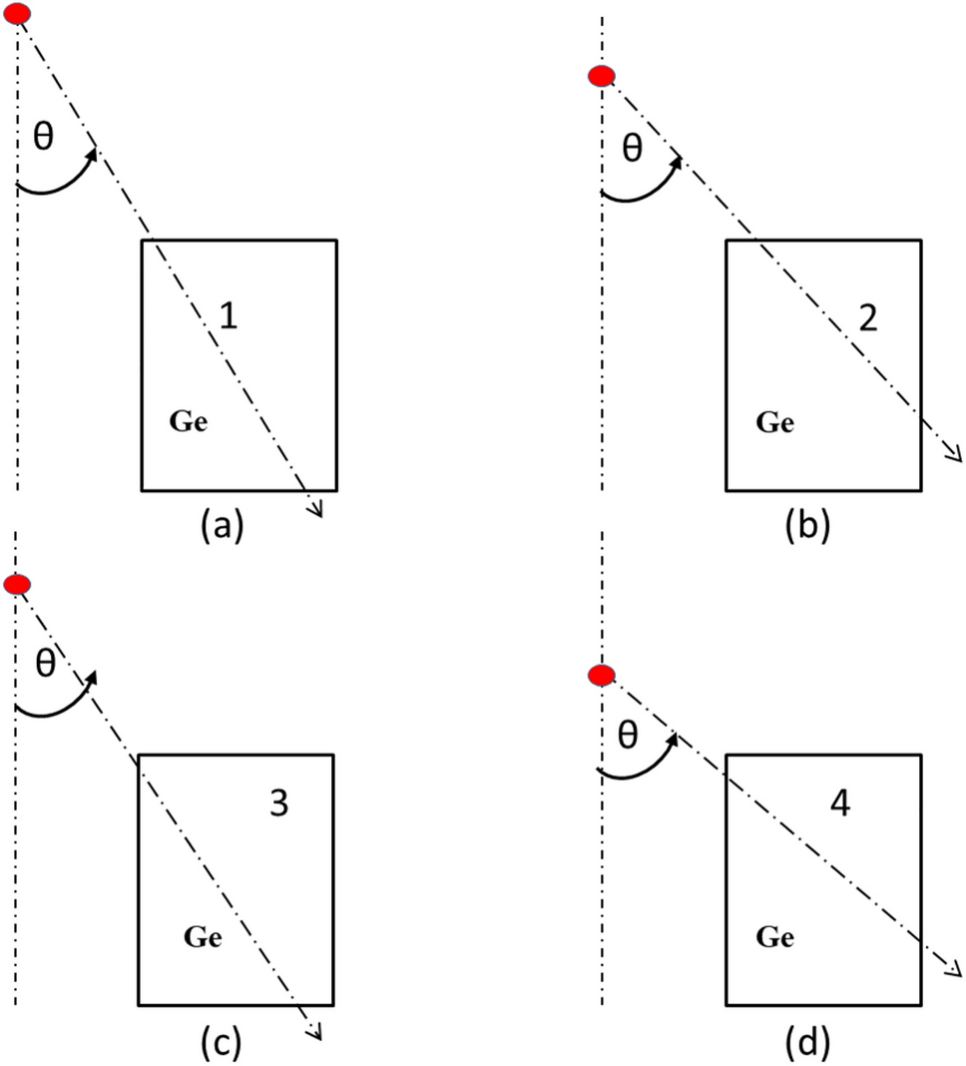
The potential path lengths of photons through the detector.

The optimization of the MC detector model is accomplished through comparison with experimental measurements, under the configuration outlined in Subsect. "Model optimization". The configuration under consideration encompasses all potential $\gamma$–ray trajectories within the detector body as described in Fig. 6, including those penetrating the upper surface and emerging from the bottom surface (case 1), or the lateral surface (case 2). It also includes trajectories penetrating the lateral surface and emerging from the bottom surface (case 3), or the lateral surface again (case 4) El-Gammal et al.^11^, El-Kourghly 203 et al.^12^.

Afterwards, the peak efficiency ($\varepsilon _{MC}$) calculated for the 185.7 keV peak of ^235^*U* isotope, obtained through MC simulations, is compared with estimates derived from experimental measurements ($\varepsilon _{exp}$) using Eq. (1).

1 $$\varepsilon _{exp}=\frac{Cr_{exp}}{S_A~M_{35}~\gamma },$$

where, $Cr_{exp}$: represents the experimental net count rate $(s^{-1})$ of the 185.7 keV peak of ^235^*U* isotopic mass $M_{35}$ with a specific activity $S_A$ $(s~g^{-1})$, and $\gamma$ emission probability. The MC model is fine-tuned using the trial-and-error method till the optimal accuracy between the MC calculated and experimentally estimated peak efficiency is less than 1 % as presented in Table 1.

**Table 1:** Comparison between MC and experimental results.

Angle ($\theta$)	0	30	60	90
Accuracy%	− 0.897	− 0.037	0.244	− 0.859

### Verification of the proposed assumption

The proposed method assumes that irregular uranium distribution is approximately equivalent to a uniform distribution for materials with a low–density matrix. To verify this assumption, the peak efficiencies $\varepsilon _R$ of different point sources randomly distributed throughout the barrel volume are calculated during the barrel rotation and averaged then compared to the peak efficiency of a uniform distribution $\varepsilon _u =8.12\times 10^{-5} \pm 0.13\times 10^{-5}$ as presented in Table 2. The relative standard deviation presented in Table 2 shows that the proposed assumption is valid with about 8% relative difference.

**Table 2:** The relative standard deviation corresponding to the random, and uniform distributions.

#. of sources	$RSD\%=(\frac{\varepsilon _U-\varepsilon _R}{\varepsilon _U})\times 100$	
	$\left( \varepsilon _R\pm \frac{\sigma }{\varepsilon _R}\right) 10^{-5}$	RSD%
1	8.80 ± 4.64	− 8.40
2	7.49 ± 1.31	7.76
6	7.88 ± 0.37	2.91
10	7.56 ± 0.68	6.93
14	7.66 ± 0.69	5.65
18	7.76 ± 0.70	4.43
22	8.15 ± 0.45	− 0.31
26	8.17 ± 0.47	− 0.57
30	8.14 ± 0.37	− 0.27

### Mathematical calibration curve

**Fig. 7:**
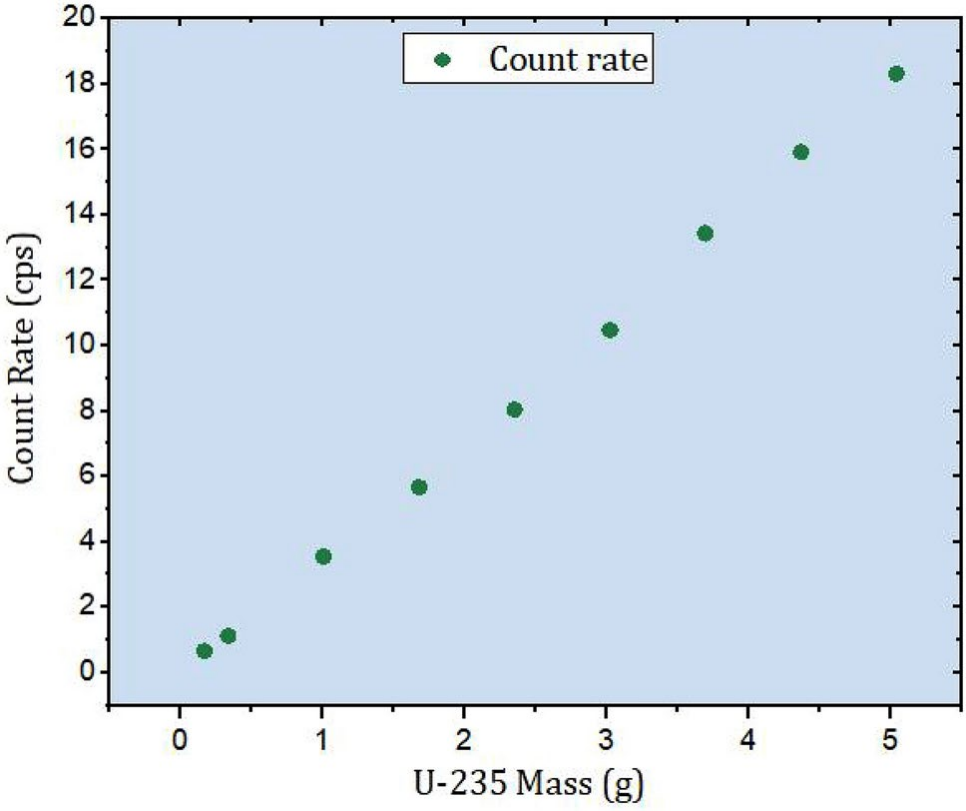
Scatter plot of the detector count rate Vs. the ^235^*U* mass.

The MC peak efficiency of point–like sources, with numbers varying from 1 to 30, randomly distributed inside a simulated barrel. This calculation is carried out while the barrel undergoes rotation from 0–${355}^0$ around its axis of symmetry, with a step size of $5^0$. The average peak efficiency ($\varepsilon _A$) is then calculated using the following Eq. (2),

2 $$\begin{aligned} \varepsilon _{A}= \frac{1}{n} \sum _{i}^{n}\varepsilon _i, \end{aligned}$$

where, $(\varepsilon )_i$ is the calculated peak efficiency corresponding to the calculation angle number *i* for a number of n rotations. Each point represent 0.168 g of ^235^*U* isotope, the MC based count rate ($Cr_{mc}$) is estimated using Eq. (3),

3 $$\begin{aligned} Cr_{mc}= S_a~\gamma _{185}~\varepsilon _A~M_{35}, \end{aligned}$$

where, $M_{35}$, $S_a$ is the ^235^*U* isotopic mass, and its corresponding specific activity, and $\gamma _{185}$ the 185.71 keV branching ratio. The datasets ($M_{35}$ and the MC–based count rate) are plotted against each other in Fig. 7 to examine potential correlations between them.

**Fig. 8:**
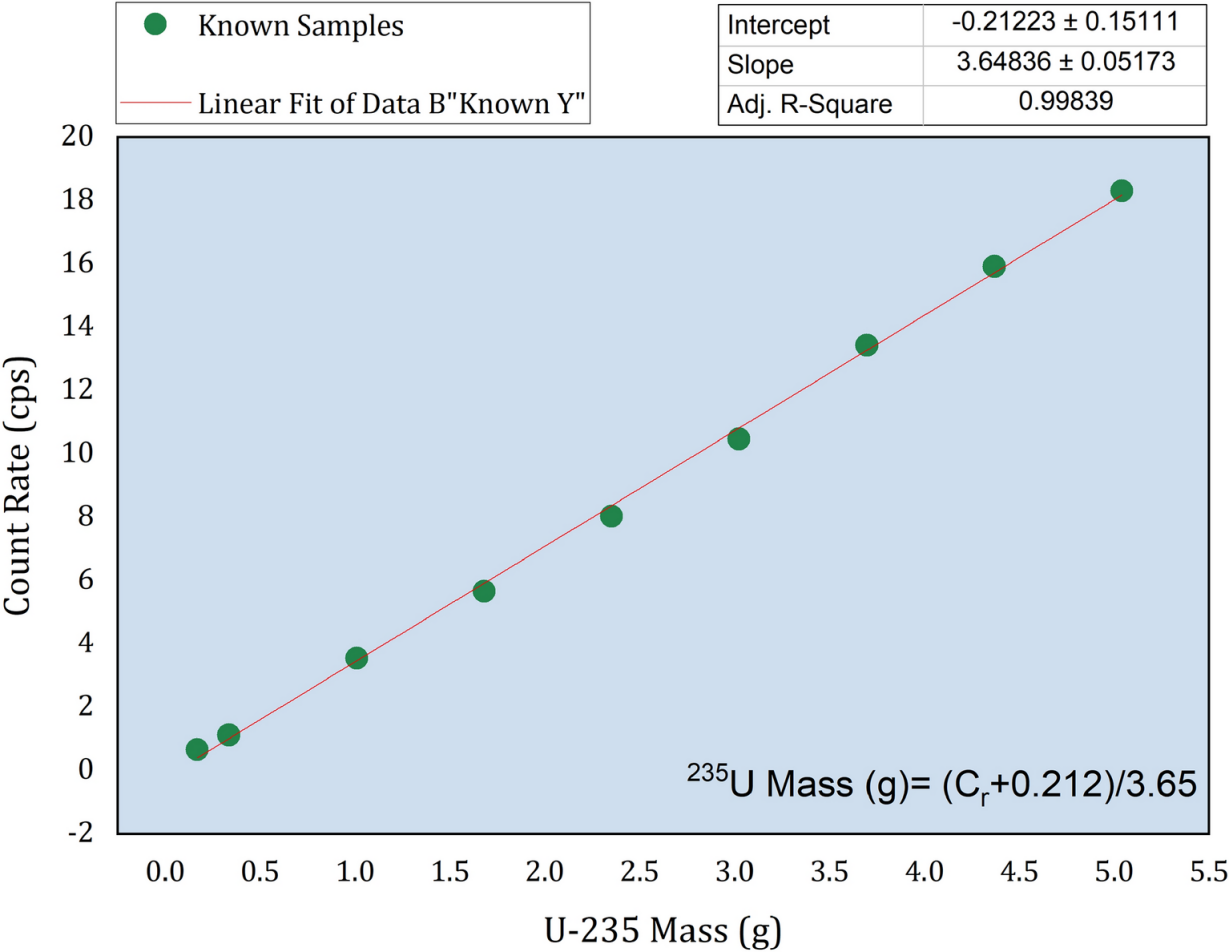
Scatter plot of the detector count rate Vs. the ^235^*U* mass and their linear fitting equation.

The plotted data sets are examined for potential outliers and points of influence to assess any leverage or bias effects on the regression line. The linear regression analysis is conducted using the ORIGIN Lab package software, as illustrated in Fig. 8.

Equation (4) defines the linear regression that characterizes the relationship between the mass of ^235^*U* and its corresponding detector response function (the count rate of the 185.7 keV $\gamma$-energy line, *Cr*).

4 $$\begin{aligned} M_{35}= \frac{Cr+0.212}{3.65}, \end{aligned}$$

In this context, the values $3.65 \pm 0.05$ for the gradient of the line and $-0.212 \pm 0.15$ for the intercept are obtained from the regression analysis, providing the best fit that minimizes the sum of squared residuals between the datasets. The regression analysis results in Fig. 8 indicate a strong correlation between the datasets, with a correlation coefficient of approximately 0.16% relative difference, indicating a very close value of the adjusted $R^2$ to unity.

#### Method validation and accuracy

To validate the proposed calibration methodology, the average net count rates of the 185.71 keV $\gamma$-energy line from distributions DR1–DR6 (as detailed in Sect. Experimental measurements) are used. These count rates are used to estimate the ^235^*U* masses based on the calibration function in Eq. (4) and the peak efficiency for a uniform distribution. The estimated ^235^*U* isotopic masses are then compared to certified values as presented in Table 3. The results indicate that the proposed methodology is valid with a maximum relative error of approximately 8 % for random distributions and 12 % for uniform distribution, which is an acceptable level of accuracy for declarations involving low quantities of concentration of NMs contained in NSW. The average deviations for random and uniform distributions are of about 3.6 % and 3.9 % respectively, confirming the validity of the introduced assumption.

**Table 3:** The relative standard deviation corresponding to the different distributions for random, and uniform.

Distribution Id	$Deviation\%=(\frac{M_{35C}-M_{35E}}{M_{35C}}) \times 100$}	
	Random	Uniform
D01	− 3.91	4.08
D02	7.61	11.94
D03	− 1.72	0.48
D04	-6.79	5.79
D05	0.28	0.41
D06	− 1.23	0.77

Furthermore, the accuracy of the proposed methodology is assessed using extreme distributions of NM throughout the barrel. The average peak efficiencies for these distributions, detailed in Sect. "Method accuracy", are compared to those of the calibration curve (random) and the uniform peak efficiency. Table 4 displays the relative errors between these results. The comparison results indicate that the proposed calibration method considering either random or homogeneous source and matrix distributions can be used for estimating the uranium mass content in NSW with an accuracy of approximately 12 %.

**Table 4:** The relative standard deviation corresponding to the different distributions for random, and uniform.

Distribution Id	Relative standard deviation %	
	Random	Uniform
BD01	− 11.31	− 8.37
BD02	8.33	2.34
BD03	− 0.16	1.46
BD04	− 2.38	− 0.75

The method assumes low-Z matrix material. Presence of unknown amounts of high-Z elements may bias the measured U content to low values.

## Conclusion

This work presents and validates a mathematical calibration approach using Monte Carlo software to estimate the uranium mass content in NSW. This waste is produced as a byproduct of uranium production, waste management, and various research and development (R&D) activities. It is characterized by a low-density matrix and contains relatively small quantities of uranium. This estimation is essential for nuclear safeguards purposes. The calibration is performed using MC calculations, assuming that the random distribution is approximately equal to the homogeneous one for low-density materials. The method’s validation is conducted by comparing it with experimental measurements. To reduce errors caused by non-uniform uranium and matrix distributions, the barrels are rotated along their symmetry axes. The method could effectively applied to estimate uranium mass content in NSW with irregular uranium and matrix distributions, achieving accuracy of about 12%.

## Data Availability

The datasets used and/or analysed during the current study available from the corresponding author on reasonable request.
